# Working towards consensus on methods used to elicit participant-reported safety data in uncomplicated malaria clinical drug studies: a Delphi technique study

**DOI:** 10.1186/s12936-017-1699-x

**Published:** 2017-01-28

**Authors:** Nyaradzo Mandimika, Karen I. Barnes, Clare I. R. Chandler, Cheryl Pace, Elizabeth N. Allen

**Affiliations:** 1 Division of Clinical Pharmacology, Department of Medicine, University of Cape Town, Cape Town, South Africa; 2 Department of Global Health & Development, London School of Hygiene & Tropical Medicine, London, UK; 3 Department of Clinical Sciences, Liverpool School of Tropical Medicine, Liverpool, UK

**Keywords:** Elicitation methods, Clinical trials, Safety, Adverse events, Non-study medication, Delphi

## Abstract

**Background:**

Eliciting adverse event (AE) and non-study medication data reports from clinical research participants is integral to evaluating drug safety. However, using different methods to question participants yields inconsistent results, compromising the interpretation, comparison and pooling of data across studies. This is particularly important given the widespread use of anti-malarials in vulnerable populations, and their increasing use in healthy, but at-risk individuals, as preventive treatment or to reduce malaria transmission.

**Methods:**

Experienced and knowledgeable anti-malarial drug clinical researchers were invited to participate in a Delphi technique study, to facilitate consensus on what are considered optimal (relevant, important and feasible) methods, tools, and approaches for detecting participant-reported AE and non-study medication data in uncomplicated malaria treatment studies.

**Results:**

Of 72 invited, 25, 16 and 10 panellists responded to the first, second and third rounds of the Delphi, respectively. Overall, 68% (68/100) of all questioning items presented for rating achieved consensus. When asking general questions about health, panellists agreed on the utility of a question/concept about any change in health, taking care to ensure that such questions/concepts do not imply causality. Eighty-nine percent (39/44) of specific signs and symptoms questions were rated as optimal. For non-study medications, a general question and most structured questioning items were considered an optimal approach. The use of mobile phones, patient diaries, rating scales as well as openly engaging with participants to discuss concerns were also considered optimal complementary data-elicitation tools.

**Conclusions:**

This study succeeded in reaching consensus within a section of the anti-malarial drug clinical research community about using a general question concept, and structured questions for eliciting data about AEs and non-study medication reports. The concepts and items considered in this Delphi to be relevant, important and feasible should be further investigated for potential inclusion in a harmonized approach to collect participant-elicited anti-malarial drug safety data. This, in turn, should improve understanding of anti-malarial drug safety.

**Electronic supplementary material:**

The online version of this article (doi:10.1186/s12936-017-1699-x) contains supplementary material, which is available to authorized users.

## Background

As part of efforts to eradicate malaria and stem the development of resistance to anti-malarial treatment, novel compounds or new molecular entities (NMEs) are being developed to meet the continued need for both the treatment and prevention of malaria [1, 2]. Before these can be marketed for use in the general population, they must first be assessed in clinical trials to establish their efficacy and safety profiles. Due to inherent limitations of clinical trials to fully assess safety, more studies are then conducted post-licensure to continue to build knowledge about adverse drug effects (and effectiveness) in real-world contexts and vulnerable populations excluded from clinical trials [3]. Pooling safety data collected during numerous clinical studies facilitates the more accurate definition of the nature and risk of adverse effects. This is especially important where the harm: benefit balance may be shifted when medicines are to be used in asymptomatic or uninfected people to prevent malaria or reduce malaria transmission. For instance, a relatively minor adverse drug reaction in a patient may be unacceptable if the drug is introduced as a disease preventive strategy in a healthy population, such as during mass drug administrations, when potentially more people with either contra-indicated or understudied conditions will be exposed [4–6].

Regulatory agencies recommend the method of collecting safety data to be explicit in study reports because of its potential impact on the study results, and, furthermore, that methods should be standardized within drug development programmes [7]. However, they do not in general specify which methods to use. This compromises the interpretation of individual studies and subsequent systematic reviews or meta-analyses [8]. Drug safety and tolerability profiles are generated by gathering adverse event (AE) data which are then considered for any potential relationship to the study drug by considering other factors, including what is known about a participant’s medical history and use of non-study medication. Objective AE assessments include medical examinations and laboratory tests, which are likely to be standardized within and between studies [9]. Studies also rely on subjective AE reports that are obtained directly from trial participants by asking them about their health status over a defined period of time. The impact of the questioning (elicitation) method on safety data is considered to be an important issue that has not received enough attention [10, 11]. More structured questioning (such as with checklists or rating scales) increases the number of AEs reported compared to an open-ended or general enquiry [12, 13]. Proponents for a general enquiry believe this collects data which is more clinically meaningful even though it may be less sensitive [14]. An argument against the structured enquiry reflects concerns that it artificially increases the AE reporting rate as it may be suggestive, thereby biasing participant responses [15]. Structured methods may also be more time consuming and less feasible to conduct in practice. Proponents of the structured enquiry, meanwhile, argue that this method is necessarily more sensitive as it detects AE data which participants do not report spontaneously [16].

For malaria specifically, a previous study conducted by this study’s authors, provides evidence that when participants forgot an AE, or did not consider it significant or relevant when asked by general enquiry, a more specific enquiry prompted a report [12]. A subsequent survey of anti-malarial drug clinical researchers found that, for capturing AEs in intervention studies, most researchers used a combination of a general and structured enquiries (31%) or structured enquiry only (26%) with fewer using a general enquiry alone (18%) [17]. A minority of researchers incorporated tools involving pictures [18]. The elicitation of previous and concomitant (non-study) medicine data reports are also important for assessing AEs for a potential relationship with a study drug. In the survey, most researchers reported using a general enquiry when asking questions about use of these with explicit reference to, for instance, “prescription only medication”, “over-the-counter medication”, “traditional medication”, “supplements” and “vaccinations”.

Following on from previous work in this area mentioned above, the aim of this research study was to seek consensus among a panel of anti-malarial drug clinical researchers about which methods they consider optimal (relevant, important and feasible) for eliciting AE and non-study medication data from participants in uncomplicated malaria drug studies. This is expected to contribute to the development of a harmonized approach that may, in turn, facilitate more accurate interpretation or pooling of participant-elicited safety data from multiple studies.

## Methods

### The Delphi

A Delphi technique study design was selected as its aim is to achieve consensus on a particular topic. This method was developed in the 1950s by the RAND Corporation and involves soliciting opinions from a panel of knowledgeable individuals with relevant experience and expertise in a particular area. Successive rounds of questioning about a topic are conducted after individual results from the previous round are reported back to the group for consideration [19]. Panellists remain anonymous to each other to avoid domination by any one individual and to allow for freedom of expression without reservation or fear of condemnation or ridicule [20–22].

Questionnaires (Additional file 1) to elicit panellist opinions were designed and pilot tested before they were administered online through SurveyGizmo^®^ so that panellists did not have to be in the same physical location, and could complete the questionnaire at a convenient time [23, 24]. In the first round, they were presented with a summary of relevant literature, including previous work by the authors, and were asked open-ended questions about what they considered the optimal method(s), concept(s) and/or approach(es) for asking study participants (or caregivers) to collect subjective AE and non-study medication data (Additional file 2). Space was provided for free-text comments and suggestions of further methods or considerations pertinent to this complex field.

Specific phrases suggested by participants in round one were then categorized into questioning concepts for rating in subsequent rounds as regards their relevance, importance and feasibility; i.e. to be optimal they should fulfil all three criteria, as all would be appropriate for malaria clinical research for detecting participant-reported AEs and non-study medication. Comments, suggestions and further methods were paraphrased into short descriptions and panellists were advised that the wording of questions or items themselves were only examples of possible phrases/words; exact terminology may be context-specific or adapted for ensuring local understanding of the question by study participants. For instance, they were asked to rate the following types of general question concepts (with examples) that may be posed to participants in an anti-malarial study: “General question about feeling (e.g. ‘How have you [has your child] been feeling?)”. Similarly, an example of a structured approach was “Questions about body parts, systems or functions (e.g. ‘Have you experienced a problem with your head, chest, breathing?’)”. This approach was applied throughout the Delphi, where appropriate. Rating was achieved using a nine-point Likert scale with options ranging from ‘strongly agree’ to ‘strongly disagree’.

In the third and final round, each panellist was sent descriptive statistics summarising all responses from round two and their individual responses. For items that had not achieved consensus they were asked whether or not they wished to change their opinion/rating in light of the summary information from all panellists [25]. The time lapse between rounds of the Delphi ranged between 3 and 6 months.

The definition of consensus in Delphi technique studies varies from study to study. For this study, each component was considered to have reached consensus when at least 70% of panellists selecting options within a three-point region indicating that most panellists disagree (score 1–3), are uncertain (score 4–6), or agree (score 7–9) that a given item is optimal [21, 25, 26]. Individual items were not mutually exclusive – for example, panellists could recommend both a general enquiry and a structured enquiry method.

### Study population and sampling

Participation was limited to individuals with experience and knowledge of clinical anti-malarial drug studies to ensure they could contribute constructively to the process [20, 24]. All panellists needed to meet one or more of the following criteria:

(i) A clinical researcher who has been responsible for a clinical trial/study where AE and/or non-study medication data was collected as part of the protocol.

(ii) An individual who has been responsible for the selection, design, review or testing of tools to collect clinical trial/study AE and/or non-study medication data.

(iii) An individual who has had direct involvement in the elicitation and recording of AE and non-study medication data from participants within malaria clinical trials/studies.

(iv) A representative of a regulatory authority responsible for reviewing clinical trial/study data, whether pre- or post-marketing.

(v) A representative of a sponsor who has funded and/or conducted malaria clinical trials.

Sampling was purposive to ensure that those who were invited met the inclusion criteria, and was largely from those who had taken part in the previous survey [17]. Individuals from organisations well known for researching or developing anti-malarial drugs were also approached. Self-selection by those invited ultimately determined who responded and participated in each of the rounds.

### Sample size

The sample size of a Delphi panel is not a statistically-bound parameter and good results can still be obtained using a comparatively small group of even heterogeneous experts [27]. Considering the highly specific inclusion criteria and anticipated attrition at each round, there was no limit set to the number who could take part, and no sample size calculation was done.

### Data management and analysis

Responses were downloaded from SurveyGizmo^®^ and analysed using Microsoft Excel^®^. The two main sections, Section A (AEs) and Section B (non-study medicines) were each further sub-divided to include three sub-sections on general questioning items, structured question items, and pictorial and/or physical questioning tool items. Consensus was assessed individually per item included in the questionnaires, and overall per sub-section.

## Results

A total of seventy-two researchers were invited to participate, of whom 25 (35%) completed round one. In round two all panellists who had completed round one were re-invited. Of these, 16/25 (64%) responded, with fifteen fully completing the round and one panellist partially completing the questionnaire. In the final round, all who had fully completed round two were invited and 10/15 (67%) responded.

### Study participants

The study population was comprised mostly of those who met at least two of the inclusion criteria. Of the 25 participants in round one, 18 (72%) had been responsible for the selection, design, review and/or testing of relevant tools, 16 (64%) had five or more years of experience in malaria clinical studies and a further 3 (12%) had 1–5 years of experience. Panellists came from seventeen countries, with over half (14/25) coming from malaria endemic countries. Most countries represented had one panellist [Australia, Bangladesh, Burkina Faso, Denmark, Gabon, The Gambia, Kenya, Malawi, Mali, Nigeria, Tanzania, Uganda, the United Kingdom] but some had more than one [USA (2), Zambia (2), Ghana (2) and Belgium (3)].

### Assessment of consensus

The percentage of all items reaching consensus overall rose from 24% in round two to 68% in round 3 (Table 1). The majority of items reaching consensus in round two were structured questions about signs and symptoms and non-study medication. See Table 2 for a summary of consensus status for individual questioning items as described below.

**Table 1:** Overall number of questions reaching consensus by sub-section

Elicitation methods	Number of items	Items reaching consensus		
		Round 2	Round 3	Total
*Adverse events*				
General questions	5	1	1	2 (40%)
Structured questions about body parts, systems or function	11	0	3	3 (27%)
Structured questions about signs and symptoms	44	16	24	40 (91%)
Pictorial questioning tools	14	0	2	2 (14%)
Other (see Table 2 for details)	8	1	4	5 (63%)
*Non*-*study medication*				
General questions	1	1	N/A	1 (100%)
Structured questions	11	5	5	10 (91%)
Pictorial and/or other physical tools	5	0	4	4 (80%)
Other	1	0	1	1 (100%)
Total	100	24	44	68 (68%)

**Table 2:** Summary of consensus status for individual questioning items

Items rated	Consensus status	
Section A		
General questions about AEs	Round 2	Round 3
Explicit question about change in health (e.g. ‘Have you observed any change or new complaint since your last visit/in the past x days [trial-specific time scale]?’)	Optimal	
Question implying causality (e.g. ‘Did your child experience any side effect from the drug since your last visit/in the past x days [trial-specific time scale]?’)		Not optimal
General question about feeling (e.g. ‘How have you [has your child] been feeling since your last visit/in the past x days [trial-specific time scale]?)		No consensus
General question about past adverse reactions to treatments (e.g. ‘Have you ever reacted badly to a drug or vaccine’)		No consensus
General question about rating any change in health (e.g. ‘How do you rate your state of health after taking the study medicine?’)		No consensus

Structured questions about body parts, systems or function (e.g. ‘Have you experienced a problem with your head, chest, breathing….?’ or ‘Have you experienced fever, headache, rash….?’)	Round 2	Round 3
Nose		Optimal
Lungs/breathing		Optimal
Head		Optimal
Ears		No consensus
Throat		No consensus
Eyes		No consensus
Chest		No consensus
Heart		No consensus
Lymphatic system		No consensus
Nervous system		No consensus
Tiredness/fatigue/weakness/lethargy		Optimal
Muscle pain		Optimal
Joint pain		Optimal
Abdominal pain		Optimal
Mood/behavioural change		Optimal
Allergic skin rash (e.g. some forms of urticaria)		Optimal
Skin abnormalities		Optimal
Dizziness		Optimal
Vision/sight problem		Optimal
Change in urine colour		Optimal
Palpitations		Optimal
Confusion		Optimal
Jaundice/icterus		Optimal
Oculogyric crisis		Not optimal
Photosensitivity (sensitivity to light)		Optimal
Spontaneous bleeding		Optimal
Constipation		Optimal
Blisters (on skin or mucous membrane)		Optimal
Hallucinations		Optimal
Headache	Optimal	
Fever	Optimal	
Cough	Optimal	
Loss of appetite	Optimal	
Vomiting	Optimal	
Nausea	Optimal	
Diarrhoea	Optimal	
Itching (no rash)	Optimal	
Peeling skin	Optimal	
Tinnitus (ringing in the ears)/hearing problem	Optimal	
Sleep disturbance/nightmares	Optimal	
Involuntary movements (e.g. rigors/convulsions/seizures)	Optimal	
Wheezing/difficulty breathing	Optimal	
Non-allergic skin rash (e.g. scabies)		No consensus
Posturing		No consensus
Pallor		No consensus
Change in walking (gait disturbance)		No consensus
*In infants*		
Eating/drinking/feeding less than normal	Optimal	
Irritable		Optimal
Abnormal sucking, if breastfed		Optimal
Crying more than normal		Optimal
Difficult to arouse	Optimal	
*In pregnant women*		
Baby movements less than normal		Optimal
Vaginal bleeding		Optimal
Increased uterine contractions, more than normal	Optimal	

Pictorial questioning tools about AEs	Round 2	Round 3
*Using photographs, drawings or pictures of the following signs and symptoms*		
Mucous membrane blisters		Optimal
Skin rash		Optimal
Headache		No consensus
Fever		No consensus
Loss of appetite		No consensus
Diarrhoea		No consensus
Jaundice/icterus		No consensus
Joint pain		No consensus
Pruritus		No consensus
*Using photographs, drawings or pictures of the following body parts*		
Respiratory system		No consensus
Gastrointestinal tract		No consensus
Central nervous system		No consensus
Skin		No consensus

Other	Round 2	Round 3
Whole body outline		No consensus
Collecting AE reports using mobile phones		Optimal
Collecting AE reports using patient diaries		Optimal
Using an archive of visual analogue scales from day 0 throughout all the follow-ups to measure potential AEs and any change in the occurrence of these events		Optimal
Openly engaging participants to discuss any concerns they have		Optimal
Keeping an archive of digital photographs of AEs	Optimal	
Collecting AE reports using group discussions		No consensus
Using flip charts, with a picture on one side for the participant and a written question for the investigator on the reverse side (to reduce investigator variability)		No consensus
Using video footage on smartphones or tablets to show how some AEs which are difficult to depict on still images may manifest e.g. seizure activity		No consensus

Section B		
General questions about previous or concomitant medication (non-study druG)	Round 2	Round 3
General questions about the use of non-study medications (e.g. ‘Have you taken any medications since your last visit/in the past x days [trial-specific time scale]?’).	Optimal	

Structured questions about previous or concomitant medication (non-study drug)	Round 2	Round 3
*Questions about the source of medicines* (e.g. ‘Have you received any medication from a traditional healer since you were last seen here?’):		
Medicine obtained from another health facility	Optimal	
Medicine obtained from a drug shop, pharmacy, chemical seller, the market (or equivalent)	Optimal	
Medicine obtained from a traditional healer, informal doctor (or equivalent)	Optimal	
Medicines already available in the home (from previous treatment courses).	Optimal	
Medicines obtained from family and/or friends		Optimal
Collecting and using naturally occurring herbs/remedies	Optimal	
*Structured questions about treatment class or specified indication*		
Analgesics/anti-inflammatory drug		Optimal
Antibiotics		Optimal
Antihistamines		Optimal
Anti-malarial		Optimal
Vitamins		No consensus

Other	Round 2	Round 3
Asking about individual treatments by name according to what is known to be locally relevant		Optimal

Pictorial and/or other physical tools to question about previous or concomitant medication (non-study druG)	Round 2	Round 3
Showing photographs or drawings of commonly used drugs or drug packets		Optimal
Showing samples of commonly used drugs or drug packets		Optimal
Showing photographs or drawings of commonly used herbs/traditional remedies		Optimal
Asking participants to bring any non-study medication they may have taken before and/or during the trial/study to scheduled visits for a physical inspection		Optimal
Showing samples of commonly used herbs/traditional remedies		No consensus

Summary of consensus status for individual questioning items rated for relevance, importance and feasibility for asking participants in uncomplicated malaria clinical trials/studies about adverse events and non-study medication

### Asking participants about AEs

Panellists agreed that an explicit question about change in health (e.g. ‘Have you observed any change or new complaint since your last visit/in the past x days [trial-specific time scale]?’) was optimal for asking about AEs during malaria clinical trials. They also agreed that a general question implying causality should be avoided (e.g. ‘Did your child experience any side effect from the drug since your last visit/in the past x days [trial-specific time scale]?’). Consensus could not be reached on items pertaining to the general questions asking about how a study participant was feeling (e.g. ‘How have you [has your child] been feeling since your last visit/in the past x days [trial-specific time scale]?’), whether they had had past adverse reactions to treatments (e.g. ‘Have you ever reacted badly to a drug or vaccine?’) or how they rated any change in health (e.g. ‘How do you rate your state of health after taking the study medicine?’).

Few specific questions about body parts, systems or functions achieved consensus about whether or not they were optimal in structured questioning (3 of 11 possible items). However, 90 percent (40/44) of specific signs and symptoms achieved consensus; 39/40 of these were rated as being optimal with one (oculogyric crisis) recommended as not optimal. The suggestion by one panellist of oculogyric crisis in round one, a complex medical term that would not be understandable to study participants, was likely to reflect a misunderstanding of the concept of or terms suitable for eliciting participant reported AEs. Consensus was also reached for showing study participants pictures or photos of ‘mucous membrane blisters’ and ‘skin rash’ to elicit reports, with no other body parts being recommended in this format. Panellists also agreed that collecting AE reports using mobile phones or patient diaries, using an archive of visual analogue scales or digital photographs of AEs and “openly engaging participants to discuss any concerns they have” were all ideas that should be considered.

### Asking participants about non-study medication use

Panellists agreed that asking a general question about the use of non-study medications (e.g. ‘Have you taken any medications since your last visit/in the past x days [trial-specific time scale]?’) was optimal, as were, in addition, all but one of the eleven structured questioning items on source of medicines, treatment class or specified indications. The one item which failed to achieve consensus was whether or not items about “vitamins” as a treatment class were optimal. Panellists could also not agree whether the option of showing samples of commonly used herbs or traditional remedies specific to the study context was optimal. However, they did agree that participants should be asked about individual treatments by name according to what is known to be locally relevant.

## Discussion

This study provides an important contribution to the methodological field of developing harmonized approaches to eliciting data about AEs and use of non-study medication within uncomplicated malaria drug studies. Consensus was achieved among panellists that a general question is optimal for capturing subjective AE reports; specifically that such an enquiry is best phrased to elicit information about a change in the participant’s health but not imply that participants should only report issues which they perceive as caused by the study drug. This is consistent with the tenet of drug safety assessment that AEs are captured regardless of any potential attribution to the drug in clinical trials [28]. While investigators have been aware of this for decades, there has been little attention to how questions are designed to help ensure that study participants report any health-related concerns but do not unwittingly filter information. The concept of an ideal phrase for general questions postulated through this Delphi study should prove useful to guide trial teams in designing their AE elicitation methods, and could be used to achieve consistency within and between trials. There was also agreement, however, between the Delphi panellists about optimal structured questions to elicit AE reports, presenting various signs and symptoms for participants to consider. General and structured questions are therefore not necessarily mutually exclusive. As shown by previous studies, a structured approach is likely to increase the sensitivity of detecting AEs, some of which may have been forgotten or not considered relevant or important by participants [11, 12]. While this may increase the workload for trial staff, using a list will ensure standard practice within and between study teams. The list developed through this Delphi study will require further refinement as there was overlap between some items identified as optimal (e.g. several skin conditions were considered for inclusion in a questioning tool individually and also as a general term). Moreover, the recommendation for using digital photos of AEs (e.g. of ‘mucous membrane blisters’**)**, which could complement a structured approach, needs to be explored in detail.

A higher degree of consensus was achieved for questions that elicit non-study medication reports compared to AE reports. There may be less controversy about whether different questioning methods impact on the data for non-study medication exposures than AEs, with a smaller potential for these questions biasing responses. These data are inherently different - AEs being subjective experiences, while taking a non-study medication a more concrete occurrence. Using structured questioning tools in conjunction with general questioning, and giving definitions and examples of individual treatments by name and treatment class according to what is relevant in the local context, will help ensure that trial participants do not inadvertently omit information based on their own understanding of what constitutes a medicine. Asking about the source of medicines could also be particularly important in communities where the use of alternative and/or traditional medicines or remedies is widespread and common practice, as previous work has shown that there may be reluctance to volunteer such traditional therapies (12). In some instances the indication for which the non-study medication was taken could identify an unreported AE which may have resulted from taking the study medication [29]. There should be discussion within a research team, therefore, as to how to encourage participants to feel comfortable enough to report items without fear of negative consequences, as reflected by the Delphi panellists’ recommendation for “open engagement with participants to discuss any concerns they may have”. This is a facet of questioning other than structure, phraseology and content that should not be dismissed as obvious or trivial. As such, there is a need for reflection on the wider literature relating to health communications, including work already conducted with clinical research staff who question malaria patients about their health [18, 30, 31]. Similar to eliciting non-study medicine reports, questions about health should reflect local understandings of illness; simple translations of a particular tool would likely not be sufficient. Further work is required to aid similar interpretation of items in different settings. Should staff paraphrase or further explain certain concepts, they should understand the rationale for asking particular types of questions to capture relevant data and achieve the trial’s safety objectives, and the potential implications of subtle changes in wording on reports elicited.

Thus, the details of optimal permutations not resolved during the Delphi and refinement of this basket of potential options, should now be taken forward for further discussions within the anti-malarial drug research community. This could be done at the time of international meetings with those interested malaria researchers, ideally joined by health communications specialists and those who have experienced malaria as a patient.

### Limitations

There is clearly more work needed to fully explore the potential for harmonization of safety data elicitation methods. The Delphi was concluded before consensus on all items could be established. While consensus for those unresolved items may have been achieved in further rounds, this is not necessarily the case and the lack of consensus on these items may indicate that those items are not widely considered optimal. This may reflect the complexity of the topic, including the composite concept used for the definition of ‘optimal’, which is likely further confounded by the attrition rate of panellists, the time-lapse between rounds, and the highly varied contexts in which the panellists conduct research; although there is no consensus, for instance, a response rate of 70% has been suggested as necessary to maintain rigour, and this Delphi achieved 64% and 67% in rounds 2 and 3 respectively [32]. However, efforts were made to reduce attrition and the time between rounds, including multiple email reminders. There was indication of some misunderstanding of the concept of “participant-reported” AEs (the inclusion of oculogyric crisis).

### Recommendations

This online Delphi technique study showed that it is possible to engage multiple researchers from around the world to collaborate on working towards the development of harmonized approaches for questioning participants about AE and non-study medication data. The results should now be taken forward for further refinement and pilot testing of harmonized tools. These will require deliberation about optimal permutations of items and the potential use of mobile phones, diaries and rating scales.

## Conclusion

The development of an accurate understanding of a drug’s safety profile is essential for all medicines, especially for those anti-malarials which are distributed widely to vulnerable at-risk populations, including uninfected persons as part of prevention strategies. The participant-reported data that contribute to drug safety assessments can be influenced by the elicitation methods used to collect this information. A harmonized approach for collecting AE and non-study medication data during uncomplicated malaria clinical studies could contribute to improving the interpretation, comparison and pooling of data from different studies. Such a harmonized approach could incorporate items that achieved consensus within this Delphi technique study. For eliciting AEs these included: a particular general question concept, structured questions about certain symptoms, use of mobile phones, diaries, visual analogue scales and photographs of symptoms, and openly engaging with participants. For eliciting non-study medication reports, these include: a general question concept; structured questions about medicine sources, treatment classes and indications, and asking about local treatments by name; showing photographs, drawings or samples of commonly used treatments, and asking participants to bring non-study medication to visits. Further discussion is needed within the malaria research community as to how to refine these options for implementation in clinical trials.

## Additional files

**Additional file 1.** Delphi questionnaires. Content of online Delphi.

**Additional file 2.** Summary of relevant literature. Literature presented to panellists prior to Delphi.
