# Supplementary material for: Working towards consensus on methods used to elicit participant-reported safety data in uncomplicated malaria clinical drug studies: a Delphi technique study
Source: Malar J. 2017 Jan 28;16:52. doi: 10.1186/s12936-017-1699-x (PMC5273807; doi:10.1186/s12936-017-1699-x)
Supplement: Supplementary file 1 — Additional file 1. Delphi questionnaires. Content of online Delphi. [file 12936_2017_1699_MOESM1_ESM.pdf]

<u>Additional file 1: Delphi questionnaires</u>

Round One Questionnaire

Working towards consensus on methods used to elicit, assess and record participant-reported adverse events data in uncomplicated malaria clinical drug trials/studies: A Delphi Process

We are interested in harmonising how uncomplicated malaria clinical research participants (or their caregivers) are asked about health and treatment-taking to collect the following types of data: medical history, adverse events and previous or concomitant medications. This interest is driven by the fact that interpreting or synthesizing results is complicated if studies use different methods for ascertaining and assessing these data. When answering the questions that follow please draw on your knowledge, experiences and opinions as well as the literature provided. Please be as candid as possible in order to add to the richness of our understanding of the different options.

* The ACT Consortium is a group of researchers conducting projects relating to the implementation of Artemisinin-based combination therapies (ACT) for malaria treatment within Africa and Asia (www.actconsortium.org). This project is funded through a grant from the Bill & Melinda Gates Foundation to the London School of Hygiene & Tropical Medicine. Ethical approval for this study was granted by the University of Cape Town Research Ethics Committee. If you have any concerns regarding your rights and welfare as they relate to this study you can contact the University of Cape Town Human Research Ethics Committee (contact details).

What will being part of this Delphi require from you?

The aim of a Delphi process is to achieve consensus about a topic. In the first round we will present an overview of the methods used generally in clinical research, and more specifically in malaria clinical drug studies, to detect, assess and record participant-reported data used for assessing harms (adverse events and previous/concomitant medications). We will then ask participants to recommend any additional approaches to obtain these data (this may involve a repeat of some of our survey questions to ensure we capture all the relevant data, including information from Delphi panellists who did not take part in the survey).

In the second round you will be asked to rank the methods/approaches in terms of their relevance, importance and feasibility in uncomplicated malaria drug studies. In subsequent rounds we will send you a summary of the group's rankings, and where your own rankings fall. You will then be able to maintain your rankings or amend them. By the final round we hope to reach consensus between the panellists about which approaches, if any, are suitable for taking forward for testing and possible harmonisation within malaria clinical studies.

As a participant you may benefit from discussion with other malaria researchers with considerable experience and expertise in this field of research. A disadvantage is that a Delphi process can be time consuming and requires commitment to complete all rounds. We have, however, kept our questions as brief as possible while still ensuring richness of the data collected. It should take between 15 - 20 minutes to complete each round of the Delphi.

Once enrolled in the Delphi your identity will be concealed from other panellists and the study investigators (a research assistant not otherwise involved will manage communications). After the Delphi you are free to reveal your identity if you desire. We ask that you complete each round of the Delphi within two weeks of receiving the initial invitation. We strongly encourage you to complete all stages to add to the richness of the data collected and rigour of the study. If, however, you decide you no longer want to participate for any reason, you are free to withdraw at any point.

If you have not read the literature review yet you can do so by clicking the link below. If you have any queries please contact the study investigators: [contact details].

If you would like to continue with the Delphi please enter your email address below and continue on to the next page. Please note that by continuing we will assume you have given consent to take part.

1. Please enter your email address below: ______________________________

<u>Section A: How to ask participants about their health to collect adverse event data</u>

Through our previous survey we found that the following general question concepts are used to ask participants (or their caregivers) about their health to collect adverse events data:

- General question about feeling.
  (e.g. '*How have you [has your child] been feeling?*')
- Explicit question about change in health.
  (e.g. '*Have you observed any change or new complaint since your last visit?*')
- Question implying causality.
  (e.g. '*Did your child experience any side effects from the malaria treatment?*')

2. Are there any other general question concepts that you consider as important and feasible for asking participants (or their caregivers) about health to collect adverse event data?

- ☐ Not applicable, I do not recommend general questions at all.
- ☐ No other general question concepts considered important.
- ☐ Yes (If yes, please describe)

In our previous survey people said that they use the following types of structured questions which offer participants (or their caregivers) options to pick from when asking about their health to collect adverse event data:

- Structured questions about malaria or non-malaria signs or symptoms, including possible expected adverse drug reactions.
  (e.g. '*Have you experienced fever, headache, skin rash?*' etc.)
- Structured questions about body parts, systems or functions.
  (e.g. '*Have you experienced a problem with your head, chest, heart, breathing?*' etc.)

For structured questions, the tools shared with us by survey participants include the following items:

Signs or symptoms (including possible adverse drug reactions):

Headache
Fever
Skin rash
Pruritus
Cough
Anorexia
Muscle pain
Weakness/Lethargy
Vomiting
Loss of appetite
Dizziness
Behavioural change
Joint pain
Abdominal pain
Allergic skin rash
Non-allergic skin rash
Itching (no rash)
Diarrhoea

Tinnitus
Hearing problem
Vision/sight problem
Palpitation
Change in urine colour
Fatigue
Confusion
Numbness
Sleep disturbance
Nightmares

Infants:
Crying more than usual
Abnormal sucking

Pregnant women:
Baby movements less than normal
Contractions more than usual
Vaginal bleeding

Body parts, systems or functions:

Ears
Eyes
Head
Chest
Nose

Endocrine
Lymphatic
Cardiovascular
Respiratory/breathing
Neurological

3. Are there any other structured question items that you consider as important and feasible for asking participants (or their caregivers) about health to collect adverse event data?

- ☐ Not applicable, I do not recommend structured questions at all.
- ☐ No other structured question considered important.
- ☐ Additional signs or symptoms. (Please describe additional signs or symptoms)
- ☐ Additional body parts, systems or functions. (Please describe body parts, systems or functions)

Through our previous survey we found that questions aided by the use of pictures or pictorial diaries are used to ask participants (or their caregivers) about their health to collect adverse events data.

For example:

- Drawing of a body outline for participants to consider.
- Photograph of a rash.

4. Please provide us with specific details of the items that you recommend should be included on pictorial questioning tools or diaries about health, for example drawing of a body outline for participants to consider, photograph of a rash etc. (Please be as exhaustive as possible).

- ☐ Not applicable, I do not recommend the use of pictorial question tools at all.
- ☐ No other pictorial question tools considered important.
- ☐ Photograph, drawing or picture of signs and symptoms, body parts or whole body outline. (Please describe the photograph, drawing or picture and/or upload an example).

Apart from general, structured or pictorial questioning methods, are there any other questioning methods or approaches that you consider important and feasible for asking participants (or their caregivers) about health to collect adverse event data?

- ☐ Yes (If yes, please describe)
- ☐ No

<u>Section B: How to ask about previous or concomitant medication</u>

In our previous survey we found that the following types of general question concepts are used to ask participants about their use of non-study medications to collect previous or concomitant medication data:

General questions about the use of medications.

For example:

- '*Have you taken any medications in the past 2 weeks/since the last visit?*'

5. Are there any other general question concepts that you consider as important and feasible for asking participants (or their caregivers) about non-study medications to collect previous or concomitant medication data?

- ☐ Not applicable, I do not recommend general questions at all.
- ☐ No other general questions considered important.
- ☐ Yes (If yes, please describe).

In our previous survey we found that the following types of structured questions are used to ask participants about their use of non-study medications to collect previous or concomitant medication data:

Questions about sources of medicine:

Health facility	Chemical sellers
Drug shop	Health worker

Pharmacy	Traditional healer

Questions about treatment classes/specific indication: Pain killer, Antibiotic, Antimalarial, Vitamins

Questions about treatment name:

Amoxcillin	Sulfadoxine/pyrimethamine (Fansidar)
Artemether/lumefantrine	Co-trimoxazole
Aspirin	Paracetamol
Amodiaquine	Quinine
Chloroquine	

6. Are there any other structured question items that you consider as important and feasible for asking participants (or their caregivers) about non-study treatments?

- ☐ Not applicable, I do not recommend structured questions at all.
- ☐ No other structured question items considered important.
- ☐ Additional sources of medicines. (Please state additional sources of medicines).
- ☐ Additional treatment classes/ specific indication. (Please state additional treatment classes/ specific indication).
- ☐ Additional treatment name. (Please state additional treatment name).

Through our previous survey we found that pictorial question tools are used to ask participants about their use of non-study medications to collect previous or concomitant medication data. Examples of these tools include:

- Photographs or drawings of drugs or drug packets
- Pictures of traditional medicines
- Pictures of mosquitoes
- Samples of commonly used drug packets

7. Please provide us with details of the items that you recommend should be included in pictorial question tools or used as examples.

- ☐ Not applicable, I do not recommend the use of pictorial question tools at all.
- ☐ No other pictorial question tools considered important.
- ☐ Photographs, drawings or pictures (Please describe the photograph, drawing or picture and/or upload an example).

Apart from general, structured or pictorial question methods, are there any other questioning methods or approaches that you consider as important and feasible for asking participants (or their caregivers) about use of non-study medications to collect previous or concomitant medication data?

- ☐ Yes (If yes, please describe).
- ☐ No

Section C: Other approaches to ensure accurate health and non-study medication reports from participants

Please consider the following approaches that may be beneficial in ensuring accurate reports:

A phrase aimed at overcoming potential barriers to reporting about health or use of non-study medications.

For example:

- *'I am interested to hear about everything even if you think it is not important.'*
- *'Do not worry about telling me about something (like using herbs), you will not get into trouble.'*

Guidance for trial staff in managing communications through another person.

For example:

- Training in when and how to include children in discussions about their health when a caregiver is present.
- Training in how to manage conversations through a translator.

8. Are there any other questioning approaches (other than those mentioned above) that you consider as important and feasible for asking participants (or their caregivers) about their health or use of non-study medications?

☐ Yes (If yes, please describe).

☐ No

Thank you! Thank you for taking the time to participate in this Delphi process. We appreciate your insights into this important and complex topic. For more information on this project, or if you are interested in collaborating with our research team, please contact [details]

Round Two Questionnaire

Thank you for completing round one of the Delphi process. We would now like to invite you to complete the second round.

In round one you and twenty-five others taking part were asked about different ways to question participants (or their caregivers) in uncomplicated antimalarial treatment studies in order to collect adverse event and non-study drug use data. We now present you with the collated suggestions from round one so that you can rate each type of question in terms of its relevance, importance and feasibility. The aim of this Delphi process is to achieve consensus on a 'menu' of harmonized or standard types of core questions to be used in a variety of uncomplicated antimalarial treatment studies.

Please keep in mind the focus of this Delphi process is safety (not efficacy), and in particular participants' subjective responses to questions about adverse events and concomitant (non-study drug) medicines. The Delphi does not consider how severity or causality of adverse events are assessed, though this may be considered in future work. The different types of questioning methods need not be mutually exclusive so you can recommend

more than one type. In future we will ask about preferred combinations for a global harmonized set of tools for eliciting adverse event (AE) and concomitant medication data.We would like to encourage you once again to complete this round of the Delphi to ensure the rigour of the study findings.

1. Please enter your email address below:

<u>Section A: Asking participants about adverse events (AEs) in uncomplicated malaria treatment studies</u>

Please rate the following types of **<u>GENERAL QUESTIONS</u>** to reflect to what extent you agree or disagree that each item is relevant and important and feasible for detecting participant-reported AE data. Please note that the questions below are only examples of possible phrases, exact terminology may be context-specific.

Please use the rating scale below in your assessment: 1 = strongly disagree, 5 = neutral, 9 = strongly agree.

2. General question about feeling (e.g. '*How have you [has your child] been feeling since your last visit/ in the past x days [trial-specific time scale]?')*

3. Explicit question about change in health (e.g. '*Have you observed any change or new complaint since your last visit/ in the past x days [trial-specific time scale]?*')

4. Question implying causality (e.g. '*Did your child experience any side effect from the drug since your last visit/ in the past x days [trial-specific time scale]?*')

5. General question about past adverse reactions to treatments (e.g. *'Have you ever reacted badly to a drug or vaccine*').

6. General question about rating any change in health (e.g. 'How do you rate your state of health after taking the study medicine?').

Please rate the following types of **<u>STRUCTURED QUESTIONS</u>** to reflect to what extent you agree or disagree that each item is relevant and important and feasible for detecting participant-reported AE data. Please note that the questions below are only examples of possible phrases, exact terminology may be context-specific.

Please use the rating scale below in your assessment: 1 = strongly disagree, 5 = neutral, 9 = strongly agree.

Questions about body parts, systems or functions (e.g. '*Have you experienced a problem with your head, chest, heart, breathing?*' etc.):

7. Ears

8. Nose

9. Throat

10. Eyes

11. Head

12. Chest

13. Endocrine system (e.g. diabetes, thyroid)

14. Heart

15. Lungs/Breathing
16. Lymphatic system
17. Nervous system (e.g. seizures, migraines)

Structured questions about signs or symptoms, including possible expected adverse drug reactions (e.g. '*Have you experienced fever, headache, skin rash etc.?*'). Some of these may be listed several times in different ways [e.g. different types of skin rash plus a summary question about skin problems]. You can indicate your preference through your rating score for each one.

18. Headaches
19. Fever
20. Cough
21. Tiredness/ fatigue/ weakness/ lethargy
22. Muscle pain
23. Joint pain
24. Abdominal pain
25. Loss of appetite
26. Vomiting
27. Nausea
28. Diarrhoea
29. Mood/ behavioural change
30. Skin rash
31. Non-allergic skin rash (e.g. scabies)
32. Allergic skin rash (e.g. some forms of urticaria)
33. Itching (no rash)
34. Peeling skin
35. Skin abnormalities
36. Dizziness
37. Tinnitus (ringing in the ears)/ hearing problem
38. Vision/sight problem
39. Change in urine colour
40. Palpitations
41. Confusion
42. Sleep disturbance/ nightmares
43. Jaundice/icterus
44. Oculogyric crisis
45. Posturing
46. Photosensitivity (sensitivity to light)
47. Spontaneous bleeding
48. Involuntary movements (e.g. rigors/ convulsions/ seizures)
49. Constipation

50. Blisters (on skin or mucous membrane)
51. Hallucinations
52. Wheezing/ difficulty breathing
53. Pallor
54. Change in walking (gait disturbance)

In studies involving young children
55. Crying more than normal
56. Abnormal sucking (if breastfed)
57. Eating/drinking/ feeding less than normal
58. Irritable
59. Difficult to arouse

In studies involving pregnant women
60. Increased uterine contractions (more than normal)
61. Baby movements less than normal
62. Vaginal bleeding
63. What is the maximum number of symptoms the participants can be reliably asked about? ________

Please rate the following types of **PICTORIAL QUESTIONING TOOLS** to reflect to what extent you agree or disagree that each item is relevant and important and feasible for detecting participant-reported AE data.

Please use the rating scale below in your assessment: 1 = strongly disagree, 5 = neutral, 9 = strongly agree.

Using photographs, drawings or pictures of the following signs and symptoms:
64. Headache
65. Fever
66. Loss of appetite
67. Diarrhoea
68. Skin rash
69. Mucous membrane blisters
70. Jaundice
71. Joint pain
72. Pruritus

Using photographs, drawings or pictures of the following body parts:
73. Respiratory system
74. Gastrointestinal tract
75. Central nervous system
76. Skin

77. Whole body outline (to mark or point to)

Other items

In round one you and other participants were asked if there were any other questioning methods or approaches considered relevant, important and feasible for asking participants about AEs apart from the general, structured or pictorial methods. The responses suggested as potentially useful depending on the context are listed below. Please rate each response to reflect to what extent you agree or disagree that each item is relevant and important and feasible for detecting participant-reported AEs in uncomplicated malaria treatment studies.

Please use the rating scale below: 1 = strongly disagree, 5 = neutral, 9 = strongly agree

78. Collecting AE reports using mobile phones
79. Collecting AE reports using patient diaries
80. Collecting AE reports using group discussions.
81. Using flip charts, with a picture on one side for the participant and a written question for the investigator on the reverse side (to reduce investigator variability).
82. Using video footage on smartphones or tablets to show how some AEs which are difficult to depict on still images may manifest e.g. seizure activity.
83. Keeping an archive of digital photographs of AEs
84. Using an archive of visual analogue scales from day 0 throughout all the follow-ups to measure potential AEs and any change in the occurrence of these events.
85. Openly engaging participants to discuss any concerns they have

Section B: Asking participants about previous or NON-STUDY (CONCOMITANT) MEDICATION in uncomplicated malaria treatment studies.

Please rate the following types of **GENERAL QUESTIONS** to reflect the extent to which you agree or disagree that each item is relevant and important and feasible to collect non-study medication data. Please note that the questions below are only examples of possible phrases, exact terminology may be context-specific.

Please use the rating scale below in your assessment: 1 = strongly disagree, 5 = neutral, 9 = strongly agree.

86. General questions about the use of non-study medications (e.g. '*Have you taken any medications since your last visit/ in the past x days [trial-specific time scale]?*').

Please rate the following types of **STRUCTURED QUESTIONS** to reflect the extent to which you agree or disagree that each item is relevant and important and feasible to collect non-study medication data. Please note that the questions are only examples of possible phrases, exact terminology may be context-specific.

Please use the rating scale below in your assessment: 1 = strongly disagree, 5 = neutral, 9 = strongly agree.

Structured questions about source of medicine (e.g. '*Have you received any medication from a traditional healer since you were last seen here?*' etc.)

87. Medicine obtained from another health facility
88. Medicine obtained from a drug shop, pharmacy, chemical seller, the market (or equivalent)
89. Medicine obtained from a traditional healer, informal doctor (or equivalent)
90. Medicines already available in the home (from previous treatment courses).
91. Medicine obtained from family and/or friends
92. Collecting and using naturally occurring herbs/remedies

Structured questions about treatment class or specified indication (e.g. '*Have you taken an antibiotic, anything for malaria, vitamins?*')

93. Analgesics/ anti-inflammatory drugs
94. Antibiotics
95. Antihistamines
96. Antimalarial
97. Vitamins
98. Please rate the following statement to reflect the extent to which you feel it is relevant and important and feasible for collecting non-study medication data. "*Participants should be asked about individual treatments by name according to what is known to be locally relevant?*"

Please rate the following types of **PICTORIAL AND/OR OTHER PHYSICAL TOOLS** [used for questioning participants about non-study (concomitant) medication] to reflect the extent to which you agree or disagree that each item is relevant and important and feasible to collect non-study medication data.

Please use the rating scale below in your assessment: 1 = strongly disagree, 5 = neutral, 9 = strongly agree.

The following pictorial questioning tools or physical samples:

99. Showing photographs or drawings of commonly used drugs or drug packets
100. Showing samples of commonly used drugs or drug packets
101. Showing photographs or drawings of commonly used herbs/traditional remedies
102. Showing samples of commonly used herbs/ traditional remedies
103. Asking participants to bring any non-study medication they may have taken before and/or during the trial/study to scheduled visits for a physical inspection

Thank You! Thank you for taking the time to participate in this Delphi process. We appreciate your insights into this important and complex topic. For more information on this project, or if you are interested in collaborating with our research team, please contact [contact details].

Round Three Questionnaire

Thank you for completing round two of the Delphi process. We would now like to invite you to complete the third round. In round two you and others taking part were presented with the collated suggestions from round

one so that you could rate each type of question in terms of its relevance, importance and feasibility. We now present you with the summary of all responses from round two, along with your individual response, and we ask that you re-evaluate your rating score taking into consideration the summary of responses from the other experts taking part. Please refer back to your individual responses from round two when completing this round. Your individual responses will be sent to you via email within twenty-four hours of receiving the invitation to complete the third round. If you have not received your individual responses yet please contact [contact details] and she will resend it to you.

For each item, we will ask you if you would like to change your rating score or maintain your original value. The aim of this Delphi process is to achieve consensus on a 'menu' of harmonized or standard types of core questions to be used in a variety of uncomplicated antimalarial treatment studies. The different types of questioning methods need not be mutually exclusive so you can recommend more than one type. Please keep in mind the focus of this Delphi process is safety (not efficacy), and in particular participants' subjective responses to questions about adverse events and concomitant (non-study drug) medicines.

We would like to encourage you once again to complete this round of the Delphi to ensure the rigour of the study findings.

1. Please enter your email address below:

We achieved consensus on some of the items in round two and we present them below. You are not asked to rate these items. Consensus was reached on all of the items listed below. This means that the panel collectively rated these items as being relevant, important and feasible for questioning participants in uncomplicated malaria treatment studies about AEs and concomitant medication using the different types of questioning methods mentioned in the Delphi. We defined consensus as having no less than 70 % of panellist selecting options which are within the same three-point region (1 - 3, 4 - 6, 7 - 9) containing the median.

Asking participants about adverse events (AEs) in uncomplicated malaria treatment studies using <u>GENERAL QUESTIONS</u>:

- Using an explicit question about change in health (e.g. 'Have you observed any change or new complaint since your last visit/ in the past x days [trial-specific time scale]?')

Asking participants about adverse events (signs and symptoms) using <u>STRUCTURED QUESTIONS</u>:
We achieved consensus on the following items:

- Headache
- Fever
- Cough
- Loss of appetite
- Vomiting
- Nausea

- Diarrhoea
- Skin rash
- Itching (no rash)
- Peeling skin
- Tinnitus (ringing in the ears)/ hearing problem
- Sleep disturbance/ nightmares
- Involuntary movements (e.g. rigors/ convulsions/ seizures)
- Wheezing/ difficulty breathing

In infants:

- Eating/drinking/ feeding less than normal
- Difficult to arouse

In pregnant women

- For Increased uterine contractions (more than normal)

Additional questioning methods or approaches for asking participants about AEs apart from the general, structured or pictorial methods:

- Keeping an archive of digital photographs of AEs.

Asking participants about previous or non-study (concomitant) medication in uncomplicated malaria treatment studies using <u>GENERAL QUESTIONS</u>:

- General questions about the use of non-study medications (e.g. 'Have you taken any medications since your last visit/ in the past x days [trial-specific time scale]?').

Asking participants about source of medicines in uncomplicated malaria treatment studies using <u>STRUCTURED QUESTIONS</u>.

We achieved consensus on the following sources of medicines:

- Medicine obtained from another health facility
- Medicine obtained from a drug shop, pharmacy, chemical seller, the market (or equivalent)
- Medicine obtained from a traditional healer, informal doctor (or equivalent)
- Medicines already available in the home (from previous treatment courses).
- Collecting and using naturally occurring herbs/remedies

Asking participants about <u>ADVERSE EVENTS</u> in uncomplicated malaria treatment studies

Please state for each of the following types of **<u>GENERAL QUESTIONS</u>** previously asked of you, whether you would like to change your rating score. The summary of responses will be presented below each question.

If you choose to change your rating, please use the rating scale below in your assessment: 1 = strongly disagree, 5 = neutral, 9 = strongly agree.

2. General question about feeling (e.g. '*How have you [has your child] been feeling since your last visit/ in the past x days [trial-specific time scale]?*')

Would you like to change your rating score?

- ☐ Yes
- ☐ No

If yes, please select your new rating score below:

3. Question implying causality (e.g. '*Did your child experience any side effect from the drug since your last visit/ in the past x days [trial-specific time scale]?*')

Would you like to change your rating score?

- ☐ Yes
- ☐ No

If yes, please select your new rating score below:

4. General question about past adverse reactions to treatments (e.g. '*Have you ever reacted badly to a drug or vaccine*').

Would you like to change your rating score?

- ☐ Yes
- ☐ No

If yes, please select your new rating score below:

5. General question about rating any change in health (e.g. '*How do you rate your state of health after taking the study medicine?*').

Would you like to change your rating score? *This question is required.

- ☐ Yes
- ☐ No

If yes, please select your new rating score below:

Asking participants about ADVERSE EVENTS in uncomplicated malaria treatment studies

Please state for each of the following types of **STRUCTURED QUESTIONS** previously asked of you, whether you would like to change your rating score. The summary or responses will be presented below each question.

If you choose to change your rating, please use the rating scale below in your assessment: 1 = strongly disagree, 5 = neutral, 9 = strongly agree.

Please note that the questions below are only examples of possible phrases, exact terminology may be context-specific.

Questions about body parts, systems or functions (e.g. '*Have you experienced a problem with your head, chest, heart, breathing?*' etc.):

6. Ears

Would you like to change your rating score?

- ☐ Yes
- ☐ No

If yes, please select your new rating score below:

7. Nose

Would you like to change your rating score?

- ☐ Yes
- ☐ No

If yes, please select your new rating score below:

8. Throat

Would you like to change your rating score?

- ☐ Yes
- ☐ No

If yes, please select your new rating score below:

9. Eyes

Would you like to change your rating score?

- ☐ Yes
- ☐ No

If yes, please select your new rating score below:

10. Head

Would you like to change your rating score?

- ☐ Yes
- ☐ No

If yes, please select your new rating score below:

11. Chest

Would you like to change your rating score?

- ☐ Yes
- ☐ No

If yes, please select your new rating score below:

12. Endocrine system (e.g. diabetes, thyroid)

Would you like to change your rating score?

- Yes
- No

If yes, please select your new rating score below:

13. Heart

Would you like to change your rating score?

- Yes
- No

If yes, please select your new rating score below:

14. Lungs/Breathing

Would you like to change your rating score?

- Yes
- No

If yes, please select your new rating score below:

15. Lymphatic system

Would you like to change your rating score?

- Yes
- No

If yes, please select your new rating score below:

16. Nervous system (e.g. seizures, migraines)

Would you like to change your rating score?

- Yes
- No

If yes, please select your new rating score below:

Asking participants about SIGNS OR SYMPTOMS (including possible expected adverse drug reactions) in uncomplicated malaria treatment studies

Please state for each of the following types of **STRUCTURED QUESTIONS** about signs or symptoms (including adverse drug reactions) previously asked of you, whether you would like to change your rating score. The summary or responses will be presented below each question.

If you choose to change your rating, please use the rating scale below in your assessment: 1 = strongly disagree, 5 = neutral, 9 = strongly agree.

17. Tiredness/ fatigue/ weakness/ lethargy

Would you like to change your rating score?

- ☐ Yes
- ☐ No

If yes, please select your new rating score below:

18. Muscle pain

Would you like to change your rating score?

- ☐ Yes
- ☐ No

If yes, please select your new rating score below:

19. Joint pain

Would you like to change your rating score?

- ☐ Yes
- ☐ No

If yes, please select your new rating score below:

20. Abdominal pain

Would you like to change your rating score?

- ☐ Yes
- ☐ No

If yes, please select your new rating score below:

21. Mood/ behavioural change

Would you like to change your rating score?

- ☐ Yes
- ☐ No

If yes, please select your new rating score below:

22. Non-allergic skin rash (e.g. scabies)

Would you like to change your rating score?

- ☐ Yes
- ☐ No

If yes, please select your new rating score below:

23. Allergic skin rash (e.g. some forms of urticaria)

Would you like to change your rating score?

- ☐ Yes
- ☐ No

If yes, please select your new rating score below:

24. Skin abnormalities

Would you like to change your rating score?

- ☐ Yes
- ☐ No

If yes, please select your new rating score below:

25. Dizziness

Would you like to change your rating score?

- ☐ Yes
- ☐ No

If yes, please select your new rating score below:

26. Vision/sight problem

Would you like to change your rating score?

- ☐ Yes
- ☐ No

If yes, please select your new rating score below:

27. Change in urine colour

Would you like to change your rating score?

- ☐ Yes
- ☐ No

If yes, please select your new rating score below:

28. Palpitations

Would you like to change your rating score?

- ☐ Yes
- ☐ No

If yes, please select your new rating score below:

29. Confusion

Would you like to change your rating score?

- ☐ Yes
- ☐ No

If yes, please select your new rating score below:

30. Jaundice/icterus

Would you like to change your rating score?

- Yes
- No

If yes, please select your new rating score below:

31. Oculogyric crisis

Would you like to change your rating score?

- Yes
- No

If yes, please select your new rating score below:

32. Posturing

Would you like to change your rating score?

- Yes
- No

If yes, please select your new rating score below:

33. Photosensitivity (sensitivity to light)

Would you like to change your rating score?

- Yes
- No

If yes, please select your new rating score below:

34. Spontaneous bleeding

Would you like to change your rating score?

- Yes
- No

If yes, please select your new rating score below:

35. Constipation

Would you like to change your rating score?

- Yes
- No

If yes, please select your new rating score below:

36. Blisters (on skin or mucous membrane)

Would you like to change your rating score?

- Yes

- ☐ No

If yes, please select your new rating score below:

37. Hallucinations

Would you like to change your rating score?

- ☐ Yes
- ☐ No

If yes, please select your new rating score below:

38. Pallor

Would you like to change your rating score?

- ☐ Yes
- ☐ No

If yes, please select your new rating score below:

39. Change in walking (gait disturbance)

Would you like to change your rating score?

- ☐ Yes
- ☐ No

If yes, please select your new rating score below:

In studies involving young children

40. Crying more than normal

Would you like to change your rating score?

- ☐ Yes
- ☐ No

If yes, please select your new rating score below:

41. Abnormal sucking (if breastfed)

Would you like to change your rating score?

- ☐ Yes
- ☐ No

If yes, please select your new rating score below:

42. Irritable

Would you like to change your rating score?

- ☐ Yes
- ☐ No

If yes, please select your new rating score below:

In studies involving pregnant women

43. Baby movements less than normal

Would you like to change your rating score?

- Yes
- No

If yes, please select your new rating score below:

44. Vaginal bleeding

Would you like to change your rating score?

- Yes
- No

If yes, please select your new rating score below:

Asking participants about ADVERSE EVENTS in uncomplicated malaria treatment studies

Please state for each of the following types of **PICTORIAL QUESTIONING TOOLS** you were previously asked about, whether you would like to change your rating score. The summary or responses will be presented below each question.

If you choose to change your rating, please use the rating scale below in your assessment: 1 = strongly disagree, 5 = neutral, 9 = strongly agree.

Using photographs, drawings or pictures of the following signs and symptoms:

45. Headache

Would you like to change your rating score?

- Yes
- No

If yes, please select your new rating score below:

46. Fever

Would you like to change your rating score?

- Yes
- No

If yes, please select your new rating score below:

47. Loss of appetite

Would you like to change your rating score?

- Yes
- No

If yes, please select your new rating score below:

48. Diarrhoea

Would you like to change your rating score?

- ☐ Yes
- ☐ No

If yes, please select your new rating score below:

49. Skin rash

Would you like to change your rating score?

- ☐ Yes
- ☐ No

If yes, please select your new rating score below:

50. Mucous membrane blisters

Would you like to change your rating score?

- ☐ Yes
- ☐ No

If yes, please select your new rating score below:

51. Jaundice

Would you like to change your rating score?

- ☐ Yes
- ☐ No

If yes, please select your new rating score below:

52. Joint pain

Would you like to change your rating score?

- ☐ Yes
- ☐ No

If yes, please select your new rating score below:

53. Pruritus

Would you like to change your rating score?

- ☐ Yes
- ☐ No

If yes, please select your new rating score below:

Using photographs, drawings or pictures of the following body parts

54. Respiratory system

Would you like to change your rating score?

- ☐ Yes
- ☐ No

If yes, please select your new rating score below:

55. Gastrointestinal tract

Would you like to change your rating score?

- ☐ Yes
- ☐ No

If yes, please select your new rating score below:

56. Central nervous system

Would you like to change your rating score?

- ☐ Yes
- ☐ No

If yes, please select your new rating score below:

57. Skin

Would you like to change your rating score?

- ☐ Yes
- ☐ No

If yes, please select your new rating score below:

58. Whole body outline (to mark or point to)

Would you like to change your rating score?

- ☐ Yes
- ☐ No

If yes, please select your new rating score below:

<u>Other items</u>

In round two you and other participants were asked to rate the additional methods or approaches suggested by some respondents as potentially useful (depending on the context), in terms of their relevance; importance and feasibility for asking participants about AEs apart from general; structured or pictorial methods or approaches.

Please state for each item below whether you would like to change your rating score. The summary or responses will be presented below each question.

If you choose to change your rating, please use the rating scale below in your assessment: 1 = strongly disagree, 5 = neutral, 9 = strongly agree.

59. Collecting AE reports using mobile phones

Would you like to change your rating score?

- ☐ Yes
- ☐ No

If yes, please select your new rating score below:

60. Collecting AE reports using patient diaries

Would you like to change your rating score?

- ☐ Yes
- ☐ No

If yes, please select your new rating score below:

61. Collecting AE reports using group discussions

Would you like to change your rating score?

- ☐ Yes
- ☐ No

If yes, please select your new rating score below:

62. Using flip charts, with a picture on one side for the participant and a written question for the investigator on the reverse side (to reduce investigator variability).

Would you like to change your rating score?

- ☐ Yes
- ☐ No

If yes, please select your new rating score below:

63. Using video footage on smartphones or tablets to show how some AEs which are difficult to depict on still images may manifest e.g. seizure activity.

Would you like to change your rating score?

- ☐ Yes
- ☐ No

If yes, please select your new rating score below:

64. Using an archive of visual analogue scales from day 0 throughout all the follow-ups to measure potential AEs and any change in the occurrence of these events.

Would you like to change your rating score?

- ☐ Yes
- ☐ No

If yes, please select your new rating score below:

65. Openly engaging participants to discuss any concerns they have.

Would you like to change your rating score?

- ☐ Yes
- ☐ No

If yes, please select your new rating score below:

<u>Asking participants about NON-STUDY MEDICATION in uncomplicated malaria treatment studies</u>

Please state for each of the following types of **<u>STRUCTURED QUESTIONS</u>** used to collect non-study medication data previously asked of you, whether you would like to change your rating score. The summary or responses will be presented below each question.

If you choose to change your rating, please use the rating scale below in your assessment: 1 = strongly disagree, 5 = neutral, 9 = strongly agree.

Please note that the questions are only examples of possible phrases, exact terminology may be context-specific.

Structured questions about source of medicine (e.g. '*Have you received any medication from a traditional healer since you were last seen here?*' etc.)

66. Medicine obtained from family and/or friends

Would you like to change your rating score?

- ☐ Yes
- ☐ No

If yes, please select your new rating score below:

Structured questions about treatment class or specified indication (e.g. '*Have you taken an antibiotic, anything for malaria, vitamins?*')

67. Analgesics/ anti-inflammatory drugs

Would you like to change your rating score?

- ☐ Yes
- ☐ No

If yes, please select your new rating score below:

68. Antibiotics

Would you like to change your rating score?

- ☐ Yes
- ☐ No

If yes, please select your new rating score below:

69. Antihistamines

Would you like to change your rating score?

- ☐ Yes

- ☐ No

If yes, please select your new rating score below:

70. Antimalarial

Would you like to change your rating score?

- ☐ Yes
- ☐ No

If yes, please select your new rating score below:

71. Vitamins

Would you like to change your rating score?

- ☐ Yes
- ☐ No

If yes, please select your new rating score below:

72. Please rate the following statement to reflect the extent to which you feel it is relevant and important and feasible for collecting non-study medication data.

"Participants should be asked about individual treatments by name according to what is known to be locally relevant."

Would you like to change your rating score?

- ☐ Yes
- ☐ No

If yes, please select your new rating score below:

<u>Asking participants about in uncomplicated malaria treatment studies</u>

Please state for each of the following types of **<u>PICTORIAL AND/OR OTHER PHYSICAL TOOLS</u>** used for questioning participants about non-study (concomitant) medication previously asked of you, whether you would like to change your rating score. The summary or responses will be presented below each question.

If you choose to change your rating, please use the rating scale below in your assessment: 1 = strongly disagree, 5 = neutral, 9 = strongly agree.

The following pictorial questioning tools or physical samples:

73. Showing photographs or drawings of commonly used drugs or drug packets

Would you like to change your rating score?

- ☐ Yes
- ☐ No

If yes, please select your new rating score below:

74. Showing samples of commonly used drugs or drug packets

Would you like to change your rating score?

- ☐ Yes
- ☐ No

If yes, please select your new rating score below:

75. Showing photographs or drawings of commonly used herbs/traditional remedies

Would you like to change your rating score?

- ☐ Yes
- ☐ No

If yes, please select your new rating score below:

76. Showing samples of commonly used herbs/ traditional remedies

Would you like to change your rating score?

- ☐ Yes
- ☐ No

If yes, please select your new rating score below:

77. Asking participants to bring any non-study medication they may have taken before and/or during the trial/study to scheduled visits for a physical inspection

Would you like to change your rating score?

- ☐ Yes
- ☐ No

If yes, please select your new rating score below:

Thank You! Thank you for taking the time to participate in this Delphi process. We appreciate your insights into this important and complex topic. For more information on this project, or if you are interested in collaborating with our research team, please contact [contact details].
