# Supplementary material for: Working towards consensus on methods used to elicit participant-reported safety data in uncomplicated malaria clinical drug studies: a Delphi technique study
Source: Malar J. 2017 Jan 28;16:52. doi: 10.1186/s12936-017-1699-x (PMC5273807; doi:10.1186/s12936-017-1699-x)
Supplement: Supplementary file 2 — Additional file 2. Summary of relevant literature. Literature presented to panellists prior to Delphi. [file 12936_2017_1699_MOESM2_ESM.pdf]

Additional file 2: Summary of relevant literature

Working towards consensus on methods used to collect participant-reported harms-related data in uncomplicated malaria clinical drug trials/studies

In order to discuss the merits and methods of harmonising the way we question antimalarial drug research participants to collect medical histories, adverse events and concomitant medication data, we have summarised some pertinent literature for prospective Delphi panellists, including our own work for this particular project.

The results of clinical research studies, syntheses of reports, and analyses of pooled individual participant harms data, are influenced by the methods used to collect such data. Different questioning methods about their health may elicit non-comparable responses from participants (Ioannidis 2006). However, there is no consensus regarding the detail of how participants (in general or for malaria) should be questioned in order to generate medical history, adverse event (AEs) or previous/concomitant medication data. For AEs there is evidence that more detailed questioning (e.g. with reference to a checklist of possible symptoms or body functions) enhances responses (Bent 2006; Greenhill 2004). However, the effect of different question methods on the nature of reports is less clear. There are concerns that detailed methods could produce a deluge of 'noise', unhelpful AEs that cannot be distinguished from background rates, and that spontaneously reported events are either more clinically meaningful or more likely to be related to a trial drug compared to placebo (Barber and Santanello 1995; Wernicke 2005).

There is a dearth of research about the way medical histories and previous/concomitant medications are elicited in clinical research, despite evidence that participants fail to report medication use when asked (Hodel 2009). In other areas of pharmacoepidemiology, including case-control or cohort studies and administrative databases, there has been methodological investigation regarding the accuracy of self-reported past medical conditions and medications, through comparison with medical or prescription records (West 2005). In those contexts, recall of medical history appears dependent somewhat on the type of condition, its significance, and a willingness to share information. Pattern-of-use is influential in recall of past medications, and indication- or medication-specific questions increase prevalence estimates compared to open-ended questions (Gama 2009).

In our own work we explored the process of participant-reported health and previous/concomitant data elicitation in malaria and HIV drug interaction trials in South Africa and Tanzania (Allen 2012). Reports obtained through different questioning types (a general enquiry, followed by checklists of possible health issues and medications, and finally an in-depth interview) were compared. We also qualitatively explored participants' experiences of illness and use of medications, and their reporting behaviour. There was an overall increase in the number of reports from general enquiry, through checklists, to in-depth interview. Using checklists and interviews appeared to facilitate recognition of health issues and medications used, and consideration of what to report. Information was sometimes not reported initially because participants either *forgot*, the event or medication had *low significance to them*, it was *considered not relevant*, or because of *perceived negative consequences of reporting*. South African inpatient malaria negative/HIV positive volunteers exhibited a "trial

citizenship", working to achieve the researchers' goals, compared to Tanzanian malaria outpatients who sometimes deferred responsibility for identifying which items to report to the trial doctor. The different trial contexts thus appeared to cultivate some specific conditions that had a role in mediating recognition, reporting and articulation of these important variables.

Participants in both sites overwhelmingly recommended that more detailed questioning (checklists or in-depth interviews) helped them to report. Investigators, meanwhile, spoke in their own focus group discussions of the challenge of eliciting comprehensive but relevant data when we will never know everything. For well-studied drugs, the focus of more detailed questioning could be on known or an anticipated risk, combined with general enquires to detect anything else. But it is a quandary whether to probe for AEs that are perhaps insignificant or irrelevant to both investigators and participants. There was, however, concern that selective detailed questioning could miss minor illness that impacts on adherence, and thus efficacy and an increased risk of malaria resistance at a population level. These clinicians, reflecting how patients may be intimidated by them despite them being at pains to be otherwise, suggested that other cadres of staff be involved to overcome barriers to reporting, whether in designing elicitation strategies (social scientists), questioning participants (nurses or social scientists) or interpreting safety results (anthropologists)

We propose, based on our own research, that some barriers to reporting may be overcome by using a checklist-type of method, while others may require a different approach (such as counselling participants to quell potential concerns about reporting). However, our work was limited to two clinical sites and therefore may not be generalizable. There may be a need for researchers to reflect on their own context when considering potential barriers to reporting and possible solutions in terms of questioning methods or approaches. To contribute to knowledge about this area we recently conducted a survey of antimalarial drug clinical researchers about the methods they used in their own studies (Allen 2012b). In this, currently unpublished, survey we included 52 responses from 25 counties; 87% working at an investigational site and 75% reporting about an interventional study. For AEs, questioning in 31% of interventional studies was a combination of general (e.g. open questions about health) and structured (e.g. reference to specific health-related items), 26% used structured only and 18% general only. No observational studies used general questioning alone. A minority of studies incorporated pictorial tools. Rationales for the questioning approach included: standardisation of assessment or data capture, specificity or comprehensiveness of data sought, avoiding suggesting a response, feasibility, and seeking to understand participants' perceptions. Most respondents considered the approach they reported as optimal, though several later reconsidered this. Combining general and structured questions about non-study drug use were considered useful for revealing and identifying specific medicines, while pictures were said to enhance reports, particularly in areas of low literacy.

References

Allen EN, Mushi AK, Massawe IS, Vestergaard LS, Lemnge M, Staedke SG, Mehta U, Barnes KIB, Chandler CIR. How experiences become data: The process of eliciting adverse event, medical history and concomitant medication reports in antimalarial and antiretroviral interaction trials. *BMC Med Res Methodol*. 2013a;13(1):140.

Allen EN, Chandler CIR, Mandimika N, Pace C, Mehta U, Barnes KI. Evaluating harm associated with anti-malarial drugs: a survey of methods used by clinical researchers to elicit, assess and record participant-reported adverse events and related data. *Mal J* 2013b;12:325.

Bent S, Padula A, Avins AL: Brief communication: Better ways to question patients about adverse medical events: a randomized, controlled trial. *Ann Intern Med* 2006;21:257-61.

Barber BL, Santanello NC: Relating spontaneous adverse experience reports to scores on a questionnaire querying tolerability. *Int J Clin Pharmacol Ther* 1995;33(11):598-604.

Gama H, Correia S, Lunet N: Questionnaire design and the recall of pharmacological treatments: a systematic review. *Pharmacoepidemiol Drug Saf* 2009;18(3):175-87. Review.

Greenhill L L, Vitiello B, Fisher P, Levine J, Davies M, Abikoff H, Chrisman A K, Chuang S, Findling R L, March J, Scahill L, Walkup J, Riddle M A: Comparison of increasingly detailed elicitation methods for the assessment of adverse events in pediatric psychopharmacology. *J Am Acad Child Adolesc Psychiatry* 2004;43(12):1488 -1496.

Hodel EM, Kabanywanyi AM, Malila A, Zanolari B, Mercier T, Beck HP, Buclin T, Olliaro P, Decosterd LA, Genton B: Residual antimalarials in malaria patients from Tanzania--implications on drug efficacy assessment and spread of parasite resistance. PLoS One 2009;4(12):e8184.

Ioannidis JP, Mulrow CD, Goodman SN: Adverse events: the more you search, the more you find. *Ann Intern Med* 2006;144(4): 298-300.

Wernicke JF, Faries D, Milton D, Weyrauch K: Detecting treatment emergent adverse events in clinical trials: a comparison of spontaneously reported and solicited collection methods. *Drug Saf* 2005;28(11):1057-63.

West SL, Strom BL, Poll C: Validity of pharmacoepidemiology drug and diagnosis data. In *Pharmacoepidemiology.* 4th edition. Edited by Strom BL. Chichester: John Wiley & Sons Ltd; 2005:709-765.
